# Gallstones Detection on Dual-Energy Computerized Tomography–Is It Ready for Real-World Use? A Retrospective Observational Study

**DOI:** 10.1097/RCT.0000000000001535

**Published:** 2023-08-03

**Authors:** Shambo Guha Roy, Vaibhav Gulati, Laura Machado Pichardo, Salama Chaker, Marion Brody, Scott Rotenberg, Reza Hayeri, Jeffrey Poot, Oleg Teytelboym

**Affiliations:** From the Department of Radiology, Mercy Catholic Medical Center, Darby PA.

**Keywords:** dual-energy CT, spectral CT, gallstone, cholelithiasis

## Abstract

**Aims:**

The aims of the study are to evaluate the performance of dual-energy computed tomography (DECT) imaging in the detection of noncalcified gallstones (GSs) and to assess its performance relative to transabdominal ultrasound (US) in identifying cholelithiasis.

**Method:**

This study is a retrospective review of radiology records and images to find all patients who had both US and DECT scans within a 6-month period and were found to have GSs. Patients who did not have GSs on US served as the control group. The CT scans were reviewed by 4 radiologists who did not have access to the US results when assessing the presence or absence of GSs on the DECT scans. In case of any discrepancies among the radiologists, the majority opinion was considered. If there was a split opinion, a fifth reviewer was consulted. The data were analyzed to calculate sensitivity, specificity, positive and negative predictive values, as well as overall accuracy and to evaluate interreader variability. The absolute Hounsfield unit (HU) differences of the GSs and bile were compared between polychromatic (PC), virtual noncontrast (VNC), and virtual monochromatic (VMC) images.

**Results:**

Considering at least 3-reader agreement, sensitivity, specificity, positive predictive value, negative predictive value, and accuracy were found to be 92%, 96%, 96%, 92%, and 94%, respectively. Individual reader sensitivity varied between 87% and 92%. There was good interobserver agreement with a Fleiss’ kappa of 0.76. Quantification of the whole data set showed that no significant difference was observed in the HU values for the stones between the PC images and the VNC images. A significant increase was observed on the 50-keV VMC images compared with the PC and VNC images. In the study group, 17% stones were visualized only on the VNC or/and 50-keV VMC images, and not on the PC images. On quantitative analysis of these cases, there was a significant increase of HU in the VNC images as compared with PC images and a significant decrease of HU in the 50-keV VMC images as compared with PC images.

**Conclusions:**

Low-keV images increase stone-bile contrast. Evaluation of cholelithiasis using VNC and 50-keV VMC images demonstrated a 14% increase in sensitivity relative to conventional CT.

Cholelithiasis is a common ailment and has an estimated prevalence of approximately 20 to 25 million in the United States.^1–3^ Although most gallstones (GSs) are asymptomatic, approximately 20% patients with cholelithiasis develop complications such as acute cholecystitis, choledocholithiasis, biliary obstruction, pancreatitis, and sepsis.^1,2,4^ There are an estimated 1.8 million ambulatory visits every year, more than 750,000 cholecystectomies being performed, and a net annual cost of greater than $6 billion directly or indirectly related to GS disease.^5^

In the United States, most GSs are composed of cholesterol and only 15% are pigmented stones.^6,7^ Given the variable composition of GSs and the lack of calcification in most stones, cholelithiasis can vary widely in density and morphology. Cholesterol stones have lower attenuation than pigment stones and often appear isodense to bile on conventional computed tomography (CT). As expected, conventional CT has a low sensitivity for GSs detection, with a maximum reported value of 80%.^8,9^ Ultrasound (US), with a reported sensitivity of 98% and a specificity of 97.7% for detection of GS, has been established as the gold standard for cholelithiasis.^10,11^ Recent evidence shows that low density occult GSs may be uncovered using DECT imaging, potentially bolstering its clinical value further.^12–14^

By using 2 different energy x-ray beams, typically 140 and 80 kVp, DECT provides valuable diagnostic information across a plethora of applications.^15^ For example, a virtual monochromatic (VMC) image reconstruction can be generated from DECT scans, which represents a scan using a theoretical x-ray beam of uniform single energy rather than a spectrum. Virtual monochromatic images exaggerate small differences in density of 2 substrates that would otherwise be missed and can highlight iodine uptake/enhancement of an inflamed gallbladder, especially at low-energy reconstructions of approximately 50 kilo electron volt (keV).^12,16^ Virtual noncontrast imaging, which seeks to subtract iodine from a scan, is another DECT technique useful in the imaging of cholelithiasis and has been demonstrated to significantly improve the conspicuity of noncalcified GSs.^14^

In this study, we sought to investigate the utility and accuracy of DECT at identifying cholelithiasis at a community hospital where DECT, including both VNC and VMC reconstructions, are routinely used in the evaluation of abdominal pain. We hypothesize that DECT can detect noncalcified GSs, which are otherwise not visualized on standard CT. Previous studies using DECT for the detection of GSs used retrospective image reconstructions, which are not feasible in routine clinical practice.^13,16^ Moreover, because of a lack of control arms in these studies, the exact sensitivity and specificity in daily clinical practice remained uncertain, which we have tried to address with this article.

## METHODOLOGY

### Patient Selection

This single-institution, retrospective study was approved by our institutional review board. We searched the radiology archive (Montage) at Mercy Catholic Medial Center (Trinity Health MA) for all US between January 2018 and June 2020 using the key words “cholelithiasis” and “gallstone,” which yielded 660 studies. They were manually cross checked in the picture archieving and communicating system (PACS) for adequate image quality and availability of DECT within 6 months from the date of US. A total of 142 patients had DECT performed within 6 months from the US. Thirty-nine patients were excluded because of inadequate or incomplete CT images. The final analysis included 103 patients in the study group with US-proven GSs and DECT within 6 months. For selection of the control group, we used the key words “no cholelithiasis” and “no gallstone” in the same time frame, which yielded 2345 US studies. Among these, 156 had DECT images available. After excluding 54 studies for poor quality or inadequate images, 102 studies were included for final analysis as control group.

### Dual-Energy Computed Tomography Protocol

All CT scans were obtained using a dual-source CT scanner (Somatom Definition Flash, Siemens Healthcare). Two different x-ray tubes were mounted in one gantry of the scanner at a 94 Å angle; 128 slices were obtained (64 sections from each of the 2 detectors) with application of a flying focal spot. The field of view was up to 50 cm in one detector (detector A) and 33 cm in the other (detector B). All scans were obtained in single portovenous phase using 140 and 100 kVp for detectors A and B, respectively, with automated dose modulation, and the following parameters: collimation, 32 × 0.6 mm; rotation time, 0.5 second; and pitch, 0.8. A nonionic contrast medium (2 mL/kg body weight; iopamidol 76%, Isovue 370; Bracco Diagnostics) was injected at a rate of 2 to 3 mL/s using a power injector (Stellant Dual, Medrad), followed by injection of 30 to 40 mL of normal saline. The tube currents were adjusted in real time by automatic dose modulation provided by the manufacturer (Care Dose4D, Siemens Healthcare). Scanning delay time was fixed at 70 seconds. A tin filter was attached to the collimator of detector A, which was operated at a high kilovoltage in the dual-energy mode.

### Dual-Energy Postprocessing and Image Reconstruction

All the DECT images were auto transferred to a postprocessing workstation (Syngo MMWP 2018, Siemens Healthcare) and then sent to PACS. Our routine reconstructed images include the dual-energy PC in axial, coronal, and sagittal planes; VNC images in axial; and 50-keV VMC in axial and iodine map in axial. All images are reconstructed at 3-mm slice thickness with 3-mm increments. The dual-energy portovenous phase images were acquired as weighted average images from both detectors as follows: 50% from the 140-kVp tube voltage and 50% from the 80-kVp tube voltage image. All image reconstruction used ADMIRE (Advanced Modeled Iterative Reconstruction) and D30f medium smooth kernel.

### Qualitative Image Analysis

All the DECT images were reviewed by 4 radiologists (J.P., R.H., S.R., M.B.) with 3, 5, 8, and 11 years of experience, respectively, who were blinded to the US results for the presence or absence of GSs. A 3:1 agreement was taken as positive or negative for GSs. In case of a 2:2 result, the fifth reader (O.T.) with 13 years of experience reviewed the study.

### Quantitative Image Analysis

Quantitative analysis of the 103 patients with proven GSs on US was performed by 1 of the 2 radiologists not blinded to stone existence (S.G.R., S.C.). Two regions of interest (ROIs) were delineated using a freehand technique, one covering the stone and the other over the adjacent bile, as far away from the stone as feasible. This procedure was repeated on the following 3 generated image sets: PC, VNC, and 50-keV VMC. The mean Hounsfield unit (HU) values were determined for all ROIs. The ROIs were drawn where the GSs was seen best. They were subsequently drawn on the corresponding slices in the other 2 sets of images.

Stone numbers were noted as single or multiple. In addition, their sizes were measured in longest dimension, in the image they were best visualized. For the multiple stones, the smallest stone size was noted.

### Statistical Analysis

Quantitative variables were expressed using means and standard deviations (SD). Mean CT attenuation of the bile and stones were compared using analysis of variance. Sensitivity, specificity, positive predictive value (PPV), negative predictive value (NPV), and accuracy for stone detection was calculated for each reader and overall, taking at least a 3-reader agreement as either positive or negative. Interobserver agreement for each study was assessed using the κ statistic. The κ value was interpreted as follows: less than 0.2, poor; 0.21 to 0.40, fair; 0.41 to 0.60, moderate; 0.61 to 0.80, good; and 0.81 to 1.00, very good. A *P* value less than 0.05 was considered statistically significant. SPSS software (version 22.00, SPSS) was used for statistical analysis.

## RESULTS

Of the 205 patients involved, 91 were male, 48 (53%) in the study group and 43 (47%) in the control. The average age was 57.2 ± 16.3 years. The patients in the study group (61.5 ± 16.3 years) were significantly (*P* < 0.01) older than the controls (53.3 ± 15.7).

Of the 205 evaluations, 193 (94%) demonstrated at least 3-reader agreement and had good interobserver agreement, as indicated by Fleiss κ coefficient of 0.76. Only 12 studies were reviewed by the fifth reader (O.T.). Considering a criterion of at least 3-reader agreement, 95 of 103 cases from the study group were reported as “positive,” endorsing a sensitivity of 92%. Four of 102 cases from the control group were, however, reported “positive” as well, resulting in a false-positive rate of 4%. None of these cases were read as positive in their original report. Only 72% of the cases (74/103) with stones were reported as positive in their original reports. Of the 95 cases with stones identified in our study group, 16 (17%) were detected only on 50-keV VMC and/or VNC images and were not visualized on PC images.

The findings endorsed a sensitivity, specificity, PPV, NPV, and accuracy of 92%, 96%, 96%, 92%, and 94%, respectively. Of the 4 readers involved in the evaluation, reader 1 had the lowest sensitivity at 87%. The other readers had sensitivities greater than 90%, with reader 3 having the highest sensitivity at 92%. Reader 4 had the highest specificity at 97%, whereas reader 2 had the lowest specificity at 80%. Readers 1 and 3 had specificities greater than 90%. The overall accuracy ranged between 87% and 94% (Table 1).

**TABLE 1:** Results of the Quantitative Analysis of the Data

	Reader 1	Reader 2	Reader 3	Reader 4	At Least 3	95% CI
Sensitivity	87	91	92	91	92	85–97
Specificity	90	80	94	97	96	90–99
PPV	90	82	94	97	96	90–98
NPV	88	90	92	92	92	86–96
Accuracy	89	86	93	94	94	90–97

CI indicates confidence interval.

**FIGURE 1:**
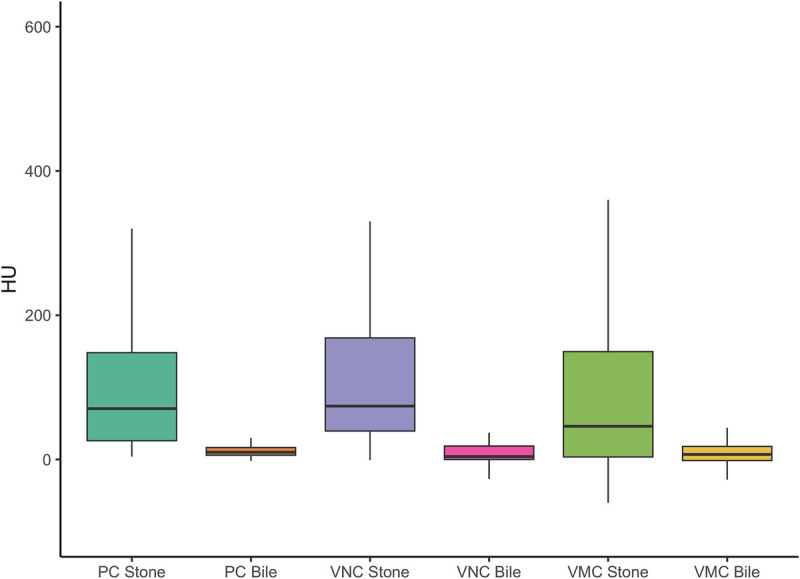
Mean HU values of GSs and bile in the 3 sets of images of the 103 patients from the study group.

On quantification, the mean HU of GSs was 157 ± 200 (range, 4 to 1082) in PC images, 134 ± 147 (range, −1 to 588) in the VNC images, and 246 ± 380 (range, −60 to 1458) in the 50-keV VMC images. The mean HU of GSs were significantly higher in the 50-keV VMC images as compared with PC or VNC images. There was no significant difference between PC and VNC images. The mean HU of bile was statistically similar in all image sets at 13 ± 10 (range, −11 to 44), 9 ± 15 (range, −27 to 55), and 9 ± 23 (range, −68 to 119) in the PC, VNC, and 50-keV VMC images, respectively (Fig. 1).

**FIGURE 2:**
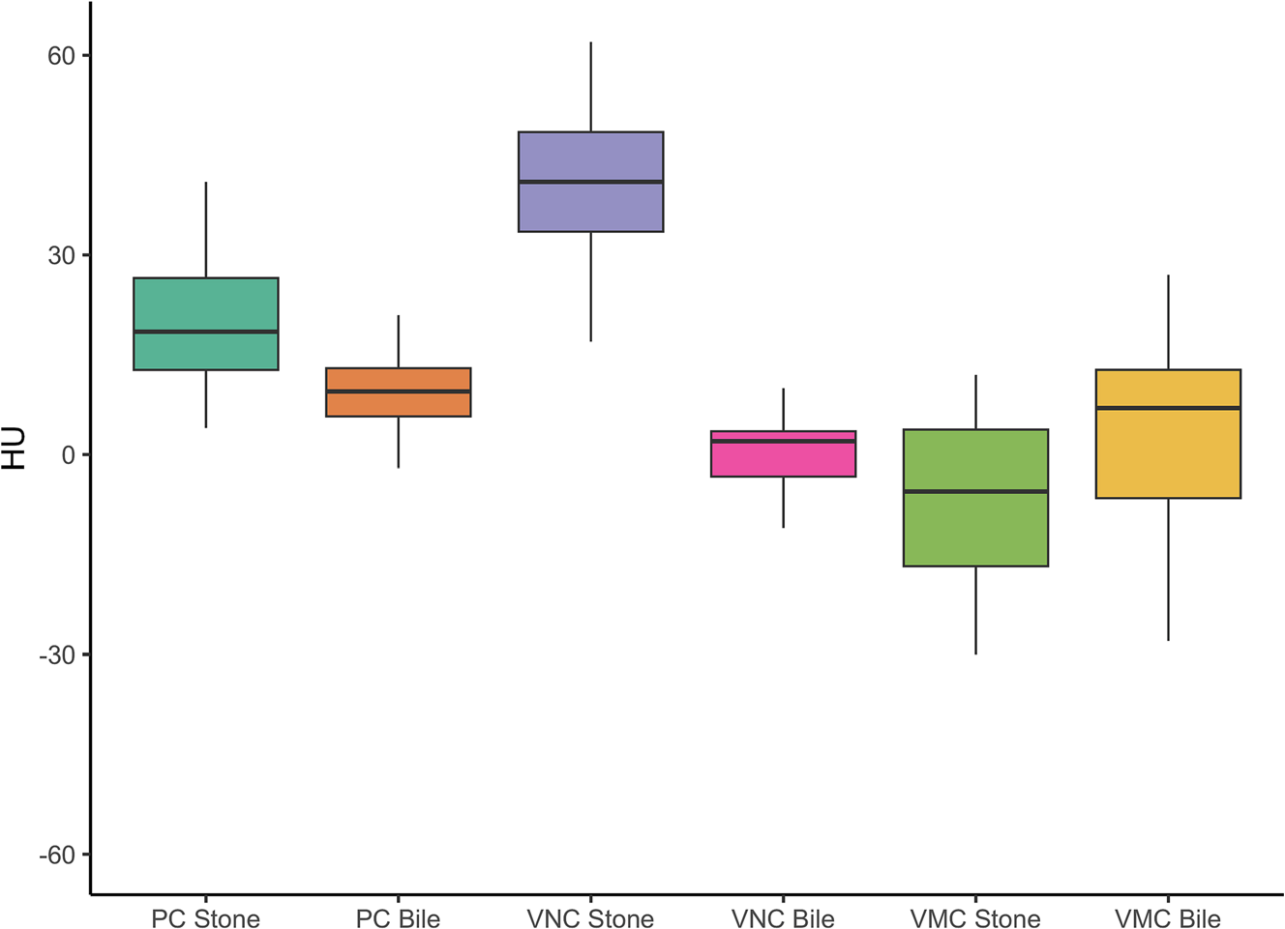
Mean HU of GSs and bile in the 3 sets of images of the subset of 16 patients in whom stones were not visually detected in the PC images.

In the subset analysis of the 16 stones, which were only visible on 50-keV VMC or/and VNC images, the mean HU increased from 20.5 ± 12.3 in the PC images to 40.5 ± 14.4 in the VNC images, which was statistically significant (*P* = 0.008), whereas the HU dropped significantly (*P* < 0.001) in the 50-keV VMC to −5.5 ± 24.7. The HU of bile was similar at 11 ± 9, 3 ± 14, and 1 ± 20 on PC, VNC, and 50-keV VMC images, respectively, in this set as well (Fig. 2).

Among the 95 cases where stones were detected on DECT, 33 were single and rest 62 patients had multiple stones. The mean stone size was 9.5 ± 6.8 mm, with maximum size of 32 mm and minimum of 1 mm. In the 16 cases where the stones were detected only on VNC and/or VMC images, the average size was 13.3 mm with the largest stone measuring 32 mm and the smallest one measuring 7 mm.

## DISCUSSION

Conventional CT, with a PC x-ray source at its heart, creates images by measuring the average attenuation of x-rays over a wide spectrum, leading to low contrast resolution. In addition, because of the considerable overlap of CT numbers for materials, conventional CT is highly limited in its ability to ascertain information on the material composition of structures. On conventional CT images, typically with energy levels between 80 and 100 keV, the CT numbers for bile and cholesterol are not statistically different, making their differentiation difficult. Baron^17^ reported a theoretical upper limit of 80% for the detection of cholelithiasis on conventional CT imaging.^16^

Spectral CT, also known as DECT, however, can get around this problem allowing for better differentiation of tissues because of their ability to measure differential attenuation of tissues at different energy levels—highlighting differences in energy-dependent absorption. The way these different energy beams are created varies from vendor to vendor: dual source–dual detector, single-source dual detector, and single-source fast switching being common types. The data obtained from the 2 energy spectra allow for the creation of material decomposition image sets, even allowing the flexibility to choose the basis material pair to represent the compositions.^16^ In addition to the creation of VNC images, VMC, and material decomposition maps, DECT allows for creation of images with higher contrast and resolution, and less artifacts, all while using less radiation compared with conventional CT to provide similar quality images.

Cholesterol stones and bile exhibit a distinct difference in their CT attenuation curves with varying energy. Previously performed studies have found that the CT number for bile is characterized by a positive value at low keV, which decreases slowly as keV is increased. On the other hand, the CT number for cholesterol GSs is predominantly negative at low keV and gradually increases with increasing keV.^13,16^ Consequently, the disparity in contrast between the stones and adjacent bile is minimal in the central range of the spectrum, where most PC images lie, leading to reduced efficacy in visualizing the GSs.^16^

Material decomposition images acquired on DECT can be used to construct fat maps, which have a high reported sensitivity for gallstones. Yang et al^16^ used a fat-based material decomposition image in their study assessing spectral CT use in the differentiation of cholesterol stones from the surrounding bile and reported a sensitivity of 95.5% and specificity of 100%. These are, however, not acquired on a regular basis and need to be reconstructed using the DECT data, requiring extra time and storage resources.

Virtual noncontrast images provide the sweet spot between DECT fat maps and conventional single-energy CT images. They are available with all DECT acquisitions, hence do not necessitate the use of any extra resources for reconstruction, and provide a substantially improved outlook for the visualization of GSs compared with PC CT images.

**FIGURE 3:**
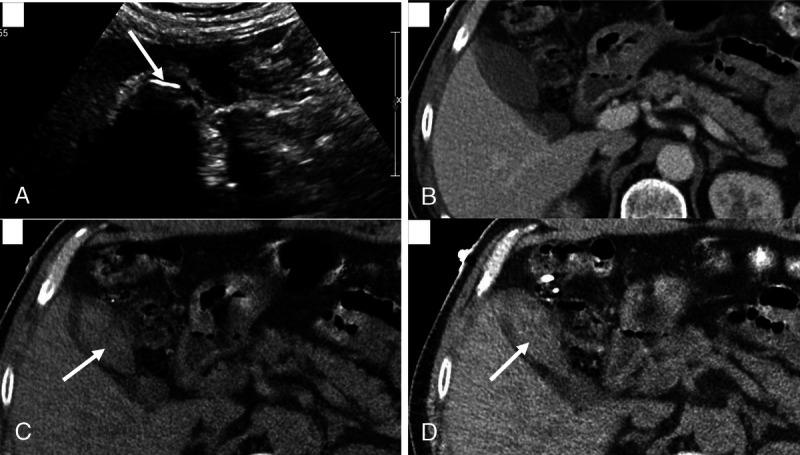
Ultrasound showing gallbladder filled with a large stone (A), which was not seen in the PC image (B), however evident on both VNC (C) and 50-keV VMC (D) images.

**FIGURE 4:**
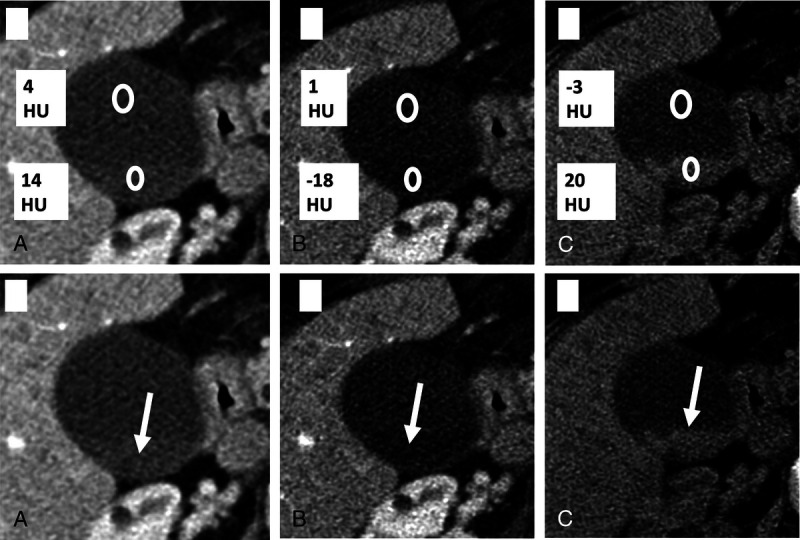
Image showing reduction in HU of the stones in 50-keV VMC images (B) and increased in HU in the VNC images (C), as compared with PC images (A). The stones are best seen on VNC images.

**FIGURE 5:**
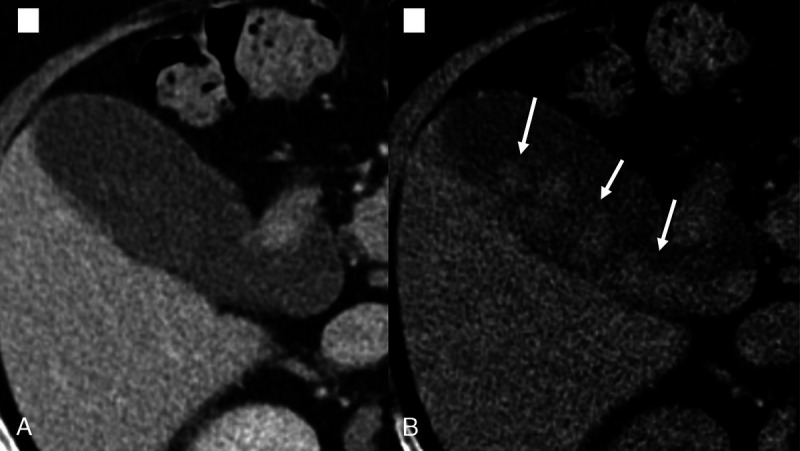
The PC images (A) show no GSs; however, they are evident on VNC images (B).

Although various reconstructions are possible in a DECT scan, almost every institute routinely uses VNC and some form of low-keV VMC reconstructions. Our study showed that routine evaluation of the VNC and VMC images from a DECT can increase sensitivity of gallstone detection by 17%, achieving a sensitivity of up to 92%. In our study, VNC images were the most helpful, which detected majority of the stones. The 16 stones, which were not visible on PC images, were best visualized on VNC images with highest contrast between the bile and the stones (Figs. 3–5). However, surprisingly, on semiquantitative assessment, there was no significant difference between mean stone HU in VNC versus PC images. Rather, there was a significant increase in mean stone HU in the low-keV VMC images. This was conflicting with our visual assessment and with existing literature, where the stone HU values are expected to decrease in the low-keV images.^13–16^

The mystery was solved by the subset analysis of the 16 patients which were not seen on the PC images. In this small subset, there was a significant increase of the HU in the VNC images and significant drop in the 50-keV VMC images as seen in (Fig. 2). The stones, which were invisible in the PC images, were mostly pure cholesterol stones and only cholesterol stones are expected to show a reduction in HU in the low-keV images. The increase in the mean stone HU in the 50-keV images seen in the overall set was due to significant increase in HU values of the calcified stones, which skewed the data.

**FIGURE 6:**
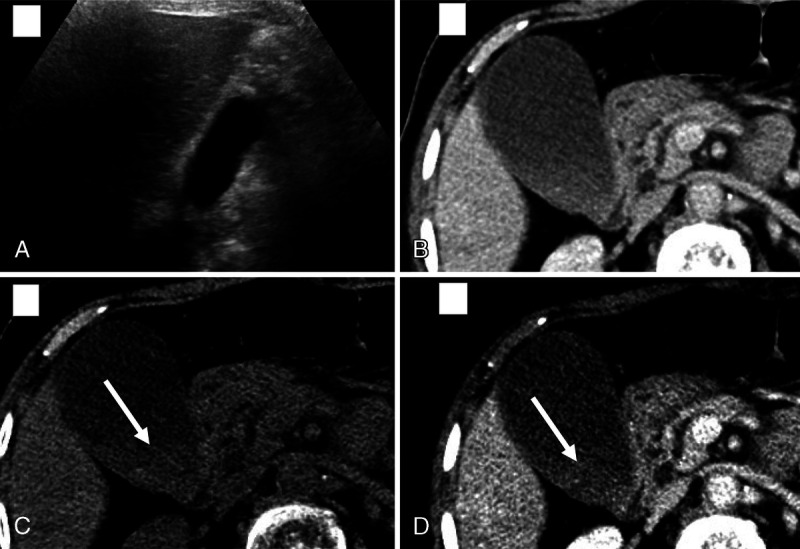
False positive: This was called as positive for GSs by all 4 reviewers based on the VNC (C) and 50-keV VMC (D) images. The PC image (B) was equivocal. Ultrasound performed (A) the same day showed no GSs.

Although both the VNC and 50-keV VMC images were always available in the PACS, only 72% of GSs were reported in the original reports. This suggests that our radiologists were not “actively” looking in the VNC and VMC images for GSs. On the other hand, the “active” search for GSs resulted in cognitive bias and resulted in 4% false-positive results, which was not present in the actual reports (Fig. 6). There is definite room for training radiologists in this regard as one of the radiologists was noted to have a false-positive rate of 20%.

Previous studies have also shown similar results. Despite the limitations in the evaluation of small stones (<9 mm^2^) or stones that are relatively radiolucent (<78 HU), VNC images offer an acceptable alternative to true nonenhanced images for the evaluation of biliary stones, causing no significant decrease in sensitivity or specificity.^18,19^ This can be especially helpful in emergency cases and can also help reduce radiation exposure to patients by eliminating the need for true nonenhanced images in these cases.^14,19^ Lee et al^14^ reported in an increase in sensitivity in the detection of cholesterol stones with VNC images, compared with true non contrast images.

**FIGURE 7:**
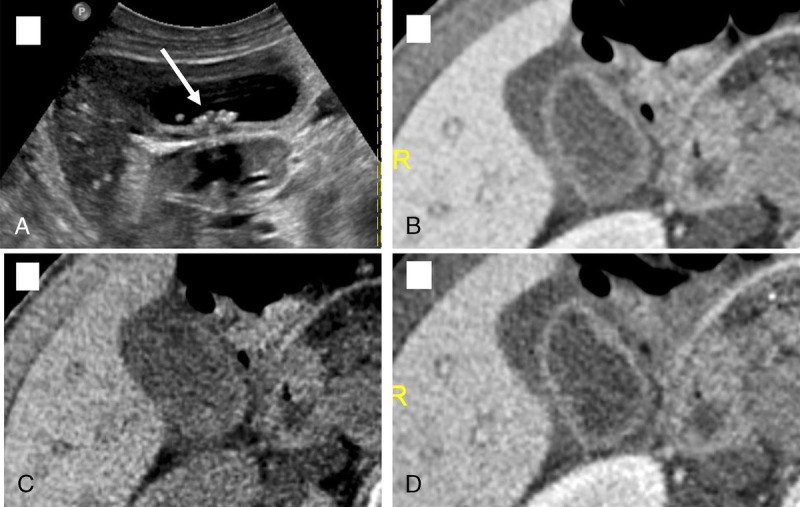
False negative: No GSs was seen by either of 4 reviewers on PC (B), VNC (C) or 50-keV VMC (C) images. Ultrasound performed (A) 2 weeks ago showed definite GSs.

Computed tomography abdomen scans are the one of the most ordered scans in emergency departments for abdominal pathologies and the volume of scans ordered continues to increase.^20–23^ Although VNC DECT images, clearly, still fail to dethrone ultrasonography for the diagnosis of GS, training radiologists to evaluate patients for GSs on VNC images could prove to be useful in reducing healthcare costs, patient wait times, and can also alleviate the need for unnecessary USs in emergency departments when GSs are suspected (Fig. 7).

Our study was restricted by several factors. The study was done with retrospective analysis involving a relatively small number of patients and only assessed cholelithiasis, not choledocholithiasis. Ultrasonography was used as the criterion standard in our analysis, and not surgery, this could potentially have led to missing some patients with GSs. The US and DECT scans were performed up to 6 months apart, and this could have led to discrepancies in their correlation. Although the radiologists were blinded to the US results, cognitive bias could not be excluded because the radiologists reading the studies were looking for GSs, which could have contributed to the higher sensitivity. It is also to be noted that the patients in the study group were significantly older than those in the control group.

## CONCLUSIONS

Dual-energy computed tomography is more effective than standard PC CT for detecting GSs.^24,25^ Our study is unique in that it evaluates the real-world sensitivity and specificity of DECT in comparison with a control group, and the results are promising. By further using DECT imaging and training radiologists to accurately detect GSs using DECT images, unnecessary US in emergency departments when GSs are suspected may be avoided, potentially reducing patient wait times and overall healthcare costs.
